# Midwives’ experiences with accompaniment service work in Norway: A qualitative study

**DOI:** 10.18332/ejm/160074

**Published:** 2023-02-24

**Authors:** Marita H. Jakobsen, Elise Udjus, Idun Røseth, Bente Dahl

**Affiliations:** 1 Centre for Women’s, Family and Child Health, Faculty of Health and Social Sciences, University of South-Eastern Norway, Borre, Norway; 2 Department of Child and Adolescent Mental Health, Telemark Hospital Trust, Skien, Norway

**Keywords:** birth, midwife, experience, qualitative, accompaniment services

## Abstract

**INTRODUCTION:**

The centralization of health services appears to be prevalent both in and outside Europe. As the distance to the nearest birth institution increases, so does the risk of unplanned births outside of an institution. A primary factor to prevent this is having a skilled birth attendant present. This study examines midwives’ experiences of working in the accompaniment services in Norway.

**METHODS:**

This was a qualitative interview study of 12 midwives working in the accompaniment services in Norway. Semi-structured interviews were conducted in January 2020. Systematic text condensation was used to analyze the data.

**RESULTS:**

The analysis identified four main themes. The midwives felt that accompaniment service work was a heavy responsibility, but it was professionally fulfilling. They felt that being on call was a lifestyle, and they were motivated by their relationships with the pregnant women. Presenting themselves as confident midwives helped the women to feel reassured. The midwives considered the cooperation within the health service to be key to good transport midwifery.

**CONCLUSIONS:**

The midwives who worked in the accompaniment services felt that their responsibility for caring for women in labor was challenging, but meaningful. Their professional knowledge was important for identifying the risk of complications and handling difficult situations. Despite carrying a heavy workload, they continued to work in the accompaniment services to ensure that women who had to travel long distances to birth institutions received appropriate help.

## INTRODUCTION

Unplanned births take place both in the home and during transport. In Norway, 459 births took place unplanned out-of-institution in 2021. In all, 170 took place in the women’s homes, 153 during transport, 11 elsewhere/unknown place, and 8 at healthcare institutions that have been shut down^1^. The number of birth institutions declined from 182 to 48 in the period 1967–2016^2^. Centralization of health services appears to be prevalent in Europe and Australia^3-5^. This has led to more out-of-institution births^3-7^. As the travel time to the nearest birth institutions increases, so does the risk of unplanned out-of-institution births^4,8^. This has proven to be a factor in increasing the risk of complications and mortality^6^. A Swedish study showed that if a woman’s travel time to the maternity ward exceeds 30 minutes, there is a greater chance that she will deliver before arrival^9^. Research also demonstrates that unplanned out-of-institution births often take place without competent maternity care^7,10,11^, even though having a skilled birth attendant present is the most crucial factor for preventing obstetric complications^6,12^.

In Norway, a 24-hour on-call service and accompaniment services are recommended for women who live more than 90 minutes away from their nearest birth institution^13^. These services are necessary for women in active labor and pregnant women with risk factors who need to be transported to a birth institution^14^. Accompaniment services must be staffed by a midwife or a doctor with adequate knowledge of obstetrics, and the services must offer good healthcare and reassurance to women in rural areas^13^. In Norway, regional health trusts are responsible for the accompaniment services. However, a survey conducted by a Norwegian newspaper in 2019 concluded that 18% of Norwegian municipalities lacked functioning accompaniment services^15^.

A few studies have examined women’s experiences with unplanned out-of-institution births. The studies of Erlandsson et al.^16^ and Vik et al.^17^ describe how women felt anxious and fearful when they realized that they would have an unplanned out-of-institution birth without a birth attendant present, but this anxiety turned into a sense of mastery and pride. Women who had an unplanned out-of-institution birth with the assistance of paramedics wanted the paramedics to act calmly in the situation^18^. They appreciated the birth attendants’ active participation, empathetic approach and good communication skills^19^. Similar results were described in a study of fathers’ experiences with unplanned out-of-institution births. The fathers highlighted the importance of having healthcare personnel, preferably a midwife, present during childbirth^20^.

There is a lack of studies exploring midwives’ experiences with accompaniment services and assisting women who have unplanned out-of-institution birth. We, therefore, conducted a study examining midwives’ experiences working in the accompaniment services in Norway.

## METHODS

### Study design, recruitment and population

The study’s design is qualitative, as such studies are suitable for describing human experiences and attitudes^21^. To recruit participants, we shared information about the project in the closed Facebook group for Norwegian midwives ‘Jordmødre i Norge’ (‘Midwives in Norway’) and via snowball recruiting^22^. We provided our contact details, which allowed midwives who were interested in participating to contact us. The main qualification criterion was previous experience with accompaniment services.

We were contacted by 14 midwives with relevant experience, and we included them using convenience sampling^22^. After conducting 12 interviews, we found that we had comprehensive and rich data that illuminated midwives’ experiences with the accompaniment services. The findings also began to be repetitive. We, therefore, decided to end the data collection. The midwives who participated in the study had worked in the profession for 3–33 years and had had different experiences with accompaniment services. Ten of these were of Norwegian ethnicity, and two were of another ethnicity but spoke Norwegian fluently. The respondents were aged 36–64 years (mean: 49) and represented seven of the eleven counties in Norway.

### Data collection

Individual semi-structured interviews were carried out in January 2020 via video calls with audio recordings. There was no time limit on the interviews, which lasted 19–85 minutes (mean: 39). The interview guide consisted of three open-ended questions, and follow-up questions were asked when necessary. First, the midwives were asked to describe their experiences with the accompaniment services as follows: ‘Could you tell me about a concrete situation in the accompaniment services you remember well?’. The participants were encouraged to share their feelings and experiences regarding the situation they described. The second question related to the midwives’ experiences working with other healthcare personnel, for example, the ambulance service. The final question was about what they wanted to share from their experiences with the accompaniment services. We ended the interview by asking each midwife how they felt discussing the topic.

### Data analysis

The interviews were transcribed and then analyzed using systematic text condensation (STC)^23^, a four-step method for thematic cross-case analysis of qualitative data. STC is inspired by Giorgi’s psychological phenomenological method, being a descriptive approach presenting the findings as close as possible to the participant’s own experience. The purpose of step 1 was to form an overall impression of the data and to identify preliminary themes in the text. In step 2, we identified meaning units and sorted them into code groups. In step 3, we formed subgroups in each code group. The content of each subgroup was summarized through condensation (i.e. an artificial quotation using the participants’ own words), written in the first-person form. Finally, in step 4, each condensed phrase was written as an analytical third-person text. Quotes were used to illuminate the results. The first and second authors were responsible for the analysis, which was discussed and revised in collaboration with all the authors.

### Ethics considerations

The study was carried out in accordance with the Declaration of Helsinki^24^. It was approved by the Norwegian Centre for Research Data (NSD) (reference no.: 836031) and assessed by the Norwegian Regional Committee for Medical and Health Research Ethics (REC no.: 76245) but was considered to be outside the scope of the Health Research Act. The participants received written information before the study began, informing them that their participation was voluntary and that they could withdraw, without giving a reason, up until the point that the data had been processed. They signed a consent form before the interview started.

## RESULTS

The analysis identified four main themes. An overview of code groups and subgroups identified during the analysis is presented in Table 1. The first theme described that midwives felt the accompaniment services to be a heavy responsibility, but at the same time, it was professionally fulfilling. Next, the midwives focused on expressing confidence to help the women feel safe. The third theme described that being on call was a lifestyle for the midwives, and they were motivated by their relationships with pregnant women. Finally, the midwives considered cooperation with other parts of the health service to be critical to good accompaniment services.

**Table 1:** Overview of code groups and subgroups (twelve Norwegian midwives’ experiences with the accompaniment services in 2020)

				
**Code groups**	Heavy responsibility, but professionally fulfilling	Women feel safe when their midwife is confident	Being on call is a lifestyle and motivated by their relationships with the pregnant woman	Cooperation is key to good accompaniment services
**Subgroups**	Responsibility Independence Motivation	Confident midwives Safe women	On call Lack of recognition Distance	Professional partners Welfare and debriefing

### Theme 1: Heavy responsibility but professionally fulfilling

Many midwives found the accompaniment service work to be challenging. They had to always be proactive and often thought about situations that might arise, and some of the situations they dealt with on their own would have been solved through teamwork at the hospital. This was described as frightening, challenging and stressful. Unforeseen events resulted in difficult decisions that required them always to know whom to contact in the health service and which resources to implement. One midwife described the sensation of being alone in a dramatic situation as follows:

*‘I was very calm, and walked down to the road where I waited for the ambulance, but when the doors opened, and I saw it right away … I felt … I felt that I was alive, to put it that way. I felt the blood just rush around my body, and even before I had examined her, I realized that things were dramatic, and I felt my heart beating. It was as if it was pounding out of my chest.’* (Interview 9)

During difficult births, some midwives noticed that they became extremely calm and emphasized that this was important. One midwife said she was relaxed about her work, even though she knew things could go wrong. The midwives were not worried that the woman would give birth, as labor often progressed quickly, without complications. They thought, nonetheless, that it was important to have a midwife present who would be able to recognize complications. Twin births, umbilical cord prolapse, premature birth, and hemorrhaging, were mentioned as situations that would be critical if no midwife working in the accompaniment services was present. In such situations, the midwives combined knowledge with their ‘gut feeling’. They stood by the decisions they made but had to be open to reconsideration. The midwives thought that they made good decisions and described their work with the accompaniment services as pleasing and rewarding but did not think anyone understood the responsibility involved:

*‘Things don’t feel hopeless because I have my knowledge. But I’m aware that it’s up to me whether it goes well or not … I feel a large lump in my belly but my head tells me I need to keep working. I’m scared, but I just have to muster enough courage to continue.’* (Interview 11)

Many midwives also worked in antenatal care and came to know the women and their families well. They found this aspect of their work to be inspiring and felt that it allowed them to be midwives in a different way. The positive feedback from the women made them feel important and that they were doing a good job. Some of the midwives liked the creative and independent way of working that the accompaniment services offered. They described the work as professionally fulfilling, allowing them to draw on the emergency care skills they had acquired during their studies. Most of the women’s labor often took place in the ambulance, and the midwife was able to sit next to the woman and offer one-to-one care throughout the journey. Some of them described childbirth as the best part of the accompaniment services:

*‘It's really fun. Because what I’ve told you might sound really awful. But the reason we keep doing this is the gratitude I’ve told you about, and because it’s such fun.’* (Interview 11)

### Theme 2: Expressing confidence to help the women feel safe

Confidence in themselves as capable practitioners was mentioned as a factor that played a major role in midwives’ job satisfaction in the accompaniment services. Experience and knowledge gave the midwives confidence. Moreover, they believed that the women needed to trust that everything would be fine, which afforded to be careful with their body language and facial expressions. The greater the complications, the more the midwives felt they had to radiate confidence and control. Some midwives felt reassured when the birth occurred in an ambulance because the paramedics could assist when necessary:

*‘I need that faith, or I would be frightened, and then the women would be able to tell and would be scared. If I were uncertain.’* (Interview 7)

The midwives found that the women trusted them and were glad they were in the ambulance with them. They knew the women and couples well from the antenatal care and tried to be accommodating. This way, the midwife knew what they needed, and the woman could dedicate her energy to the labor. The midwives felt that they were in the ambulance to provide support. This could take the form of eye contact, physical touch, breathing techniques, or simply being a calm and reassuring person. One midwife said that if a woman were in labor in the ambulance, she would seem calm on the outside, but her heart would be racing on the inside. When they arrived at the birth institution, the midwives tried to ensure that the hand-over was a good experience. If the woman felt insecure, the midwife sometimes stayed with her until she had given birth or until the woman felt secure with her new midwife:

*‘So sometimes … it’s a bit, oh, do I have to go with her? (Laughs) But you want to – you think about the pregnant woman, their birth experience. You have to remember that you’re part of their labor. And how a woman wants to be cared for during childbirth is very, very important.’* (Interview 10)

### Theme 3: Being on call is a lifestyle and is motivated by the relationship with the pregnant woman

Several midwives considered the accompaniment services a lifestyle because they could suddenly be called out to work, even when they were not on shift. One midwife said she spent half of her life on call, but her salary did not reflect this. She thought it would have been easier to handle the work if their pay reflected the on-call nature of the work. She described how being unable to move around freely felt like being shackled. The midwives brought their phones with them everywhere, even to the toilet. Their bodies told them they were on call, even if they spent a great deal of time at home. One midwife said that it took her a long time to recover from 24-hour shifts and that she felt unwell. Others reported two or three trips in a row, where a single trip could take 8 hours:

*‘The way things are now, I wonder how long I will be willing to keep this up. I mean who can be bothered doing this type of work with this pay and these terms.’* (Interview 1)

The midwives felt particularly guilty if they were on sick leave because this affected pregnant women throughout the entire region. They refused to switch off their duty phone and saw that they were seldom replaced by temporary staff. They felt that the management did not understand the importance of the accompaniment services, and they felt undermined and taken for granted. Their employer would tell them to get a new job if they were dissatisfied, but they stayed in the job because they wanted to improve the women’s situation. They talked about their professional pride and how dedicated they were to the work they did for women in labor, babies and families. This was why the management’s lack of understanding of the importance of their work infuriated the midwives:

*‘Lives will be lost! There are several examples of how lives could have been lost during the eight years we have worked if we had not been here. And that’s a sad thought!’* (Interview 8)

According to the midwives, women living in rural areas were entitled to high quality maternity care. They were at risk of complications, and using the ambulance as a ‘mobile delivery room’ was no option. They knew how quickly normal labor could turn into a high-risk birth, and having on-duty staff and accompaniment services provided safety for the entire family. The midwives reported many thoughts and stress related to factors beyond their control, such as great distances and difficult weather conditions. One midwife mentioned closed mountain passes, planes that were unable to land and cancelled ferries. What could a midwife do when a woman experiencing contractions called and she was 250 km away from the hospital:

‘Should we send them down on a hospital transport bus so they give birth at a specific time, then take the bus home again?’ (Interview 3)

### Theme 4: Cooperation is key to good accompaniment services

One midwife talked about difficult handovers at the maternity ward. There had been times when women did not want her to leave, and she thought it was unpleasant to hand over the women to a busy ward. Others felt that the handover went well, especially when they were able to deliver the baby in the hospital. The midwives often travelled with the women to the hospital early to avoid their giving birth during transport and felt that the local hospital staff understood this. Many described a good working relationship with the local hospital and felt that they trusted the midwives’ assessments. However, at larger hospitals, the midwives felt that consideration was not taken of distances and that all sorts of situations could arise within a short time during labor. They reported that the cooperation with the paramedics was good and that they felt like a team. They felt that the paramedics were pleased when they were there, and most of the midwives wanted the paramedics to be involved:

*‘I think that the cooperation with the ambulance service is very good. They’re fantastic people to work with. They’re so grateful to have a midwife along with them.’* (Interview 1)

Sometimes the midwives who worked with accompaniment services experienced difficult situations where they thought things would go wrong. In such cases, they discussed the events with the other midwives. They said this worked well and thought they learned a great deal from this. Some of them felt very much left to their own devices and did not receive any follow-up from their employer unless they experienced an extreme situation. Some of them said that they did not feel that their employer took care of them, while others reported that the management understood that accompaniment service was a special job. They had a set follow-up and debriefing procedure:

*‘It’s important to have this network, and for everyone to follow the same procedures as you. […] Because we can’t just pull a cord or push a red button if something happens. We have to handle it ourselves! So it’s very important for the people who work with this to have a network. Um, and you can’t find that network at hospital. You find it out in rural areas or in the people who work with this.’* (Interview 4)

## DISCUSSION

The midwives in our study felt that accompaniment service was a heavy responsibility that required them to make many difficult decisions on their own. Overall, they experienced the decisions they made to be sound. They were generally not worried that the women would give birth during transportation, and most reported that labor often progressed quickly without complications. Yet, the midwives perceived the potential risk of unforeseen complications as frightening, challenging and stressful, impelling them to mentally prepare by considering different scenarios that could arise. Similar results have been described in comparable studies on the experience of paramedics^25^.

The midwives’ preparedness for complications are not unfounded, as different studies report inconsistent findings about the level of risk for complications for women who have an unplanned out-of-institution birth. Some studies found low risk^16^, while others found an increased risk^6,11,26,27^.

Many of the midwives in our study trusted their competence and felt confident in their decisions. They were also aware of the importance of being and presenting themselves as confident and in control to reassure the women during transport. Laboring women want a confident midwife who listens to them, provides information and includes them. This is highlighted by research which shows that the quality of the midwives’ presence is more important to women than the acts they perform^28^. The midwives described that a large part of labor often took place in the ambulance and that they had close contact with the women. They found that the women were glad to have them there, and they often knew them from antenatal care. Several studies indicate that women who have an unplanned out-of-institution birth feel uncomfortable, vulnerable and stressed without a midwife in the ambulance^16^. Women who receive one-to-one care from a midwife during labor say that this gives them a safe and good childbirth experience^29,30^. This also applies to women giving birth in an ambulance assisted by a midwife^20^.

In caseload midwifery (CM), women receive their ante-, intra- and postnatal care from one midwife^31^. They work independently, and often they have 24-hour shifts. This results in feelings of increased responsibility for clinical assessments and choices, especially in the event of complications^32^. Our study shows similar experiences. Despite the heavy responsibility, the midwives described the job as meaningful and professionally fulfilling. They were motivated to stay in the job due to its importance for women and the gratitude that the women showed them. Similar findings are described in CM, where the midwives are dedicated to their work and describe the satisfaction of doing a good job and being valued by the women as their ‘reward’^32,33^.

The midwives considered accompaniment service work a lifestyle due to its unpredictability and on-call nature. Research on CM supports these findings, where undefined working hours and on-call duty, which are difficult to combine with home and family life, are described as negative factors^32,33^. There is a balancing act between the advantages of the meaningful nature of the work and the disadvantages of being on call. Researchers believe that dedicated midwives who find satisfaction in delivering a high standard of work are best equipped to deal with such work structures^32,33^. In Denmark, the midwives concluded that the positive aspects of CM outshone the negative ones^33^. Such dedication to one’s work is not necessarily wholly positive; midwives’ willingness to give women excellent care can be so tiring that they ultimately leave the profession^34^. The midwives found that transport to the nearest birth institution was incredibly important for patient safety. The ambulance was important in providing good transport, but it should not serve as a ‘mobile maternity unit’. Research demonstrates that negative outcomes of unplanned out-of-institution births caused by long travel times deserve more attention^6^.

All the midwives described the cooperation with the paramedics as good. A study of paramedics’ experiences with out-of-institution births showed that they feel that ‘everything’ is fine if they bring a midwife. Nonetheless, a few paramedics report poor communication and a low level of inclusion in the cooperation with the midwives^35^. Another study on the same topic showed that paramedics felt that midwives did not give them enough feedback about the work they do^25,35^. In this study, most of the midwives wanted paramedics present to help them, as well as to give the paramedics experience. A few midwives preferred working independently, as they felt competent enough to manage independently. The midwives felt that they needed more follow-up from the management. This was why many of them wondered how long they would manage to remain in the profession. Within the accompaniment services, all the midwives in our study felt that the cooperation was good when several midwives were in a rural area. They supported each other well and discussed cases together because they were the only ones who understood each other’s experiences. The midwives who worked in CM reported the same, saying that they shared strong bonds with their colleagues^32,33^. The midwives occasionally felt isolated because they rarely worked with others^32^. Having someone to talk to was important – especially in difficult situations^33^. This was confirmed by the midwives in our study, who said that they felt very lonely without other midwives as colleagues. Research from New Zealand shows midwives decided to leave CM due to a lack of professional, social and emotional support from other midwives and the management^34^.

### Limitations

Focusing on our preconceptions is important because it affects how questions are asked and how narratives are understood. Three of the authors of this study are midwives, the fourth is a psychologist. Two are researchers, and one author has contributed previous research about unplanned out-of-hospital births.

We chose a qualitative method, well suited to illuminate an unexplored topic. It was crucial to find participants with experience from the accompaniment services for our study. The midwives who signed up for the study lived in different parts of the country, and video calls were a good tool for data collection in such circumstances. The midwives had different experiences and working conditions, depending on where they lived. This resulted in varying findings, which gave a nuanced picture. Some of the experiences shared by the informants had happened several years ago, which naturally may have affected the data and the internal validity^23^. Only midwives employed full-time in transport midwifery signed up and were included in the study. Some of the midwives expressed having an agenda for sharing their opinions regarding the negative aspects of the organization of the accompaniment services. We cannot rule out the possibility that midwives with negative working experiences from the accompaniment services were more inclined to participate in the study. This is a weakness of convenience sampling.

The study presents different perspectives from a wide range of midwives, which may increase the credibility and transferability of the study^23^. Our study included two participants who were not of Norwegian ethnicity, but all the participants had experience working in Norwegian health care, nonetheless, and spoke Norwegian fluently.

## CONCLUSIONS

This study shows that midwives experience accompaniment services as a heavy responsibility and a professionally fulfilling work. Always having to be proactive and imagine different scenarios, including potential complications that might arise, was challenging, and the midwives underscored the importance of being confident, and expressing their knowledge and expertise to reassure the women during transport. They reported many disadvantages to be on call and experienced that the management needed to understand the burdens inherent in their work. Despite dealing with difficult situations and carrying a heavy workload, they continued to work in the accompaniment services to ensure that women who must travel long distances to birth institutions receive good professional care. Having a skilled midwife present during long-distance travel is an important factor in preventing obstetric complications. However, research is needed to explore what would be necessary when organizing these services from midwives’ perspectives.

## Data Availability

The data supporting this research cannot be made available for privacy or other reasons.
